# Knock-Down of *CsNRT2.1*, a Cucumber Nitrate Transporter, Reduces Nitrate Uptake, Root length, and Lateral Root Number at Low External Nitrate Concentration

**DOI:** 10.3389/fpls.2018.00722

**Published:** 2018-06-01

**Authors:** Yang Li, Juanqi Li, Yan Yan, Wenqian Liu, Wenna Zhang, Lihong Gao, Yongqiang Tian

**Affiliations:** ^1^Beijing Key Laboratory of Growth and Developmental Regulation for Protected Vegetable Crops, College of Horticulture, China Agricultural University, Beijing, China; ^2^College of Horticulture, Henan Agricultural University, Zhengzhou, China

**Keywords:** nitrate, transporter, cucumber, *CsNRT2.1*, root growth

## Abstract

Nitrogen (N) is a macronutrient that plays a crucial role in plant growth and development. Nitrate (${\mathrm{NO}}_{3}^{-}$) is the most abundant N source in aerobic soils. Plants have evolved two adaptive mechanisms such as up-regulation of the high-affinity transport system (HATS) and alteration of the root system architecture (RSA), allowing them to cope with the temporal and spatial variation of ${\mathrm{NO}}_{3}^{-}$. However, little information is available regarding the nitrate transporter in cucumber, one of the most important fruit vegetables in the world. In this study we isolated a nitrate transporter named *CsNRT2.1* from cucumber. Analysis of the expression profile of the *CsNRT2.1* showed that *CsNRT2.1* is a high affinity nitrate transporter which mainly located in mature roots. Subcellular localization analysis revealed that CsNRT2.1 is a plasma membrane transporter. In N-starved *CsNRT2.1* knock-down plants, both of the constitutive HATS (cHATS) and inducible HATS (iHATS) were impaired under low external ${\mathrm{NO}}_{3}^{-}$ concentration. Furthermore, the *CsNRT2.1* knock-down plants showed reduced root length and lateral root numbers. Together, our results demonstrated that CsNRT2.1 played a dual role in regulating the HATS and RSA to acquire ${\mathrm{NO}}_{3}^{-}$ effectively under N limitation.

## Introduction

Nitrogen (N) is considered to be one of the most important macro-elements limiting plant growth in most agricultural systems. Millions of metric tons of N fertilizer are applied worldwide annually to increase crop or fruit yields (Good et al., 2004). However, the N use efficiency decreased from 68 to 47% in the past 50 years (Lassaletta et al., 2014), and more than half of N lost into the environment. Furthermore, high application rates of N fertilizer often result in soil and groundwater pollution (Tilman et al., 2002). Both inorganic and organic N can be absorbed by plants, but inorganic forms of N, such as nitrate (${\mathrm{NO}}_{3}^{-}$) and ammonium (${\mathrm{NH}}_{4}^{+}$), are predominate in agricultural systems. In general, ${\mathrm{NO}}_{3}^{-}$ has much higher value compared with ${\mathrm{NH}}_{4}^{+}$ in aerobic agricultural soils (Wolt, 1994). Therefore, it is important to study the mechanisms of ${\mathrm{NO}}_{3}^{-}$ uptake by plants from the soil to improve plant growth and prevent negative effects of N fertilizers on the environment.

Since ${\mathrm{NO}}_{3}^{-}$ concentration in the soil varies drastically due to the influence of soil type, soil temperature and microbial activity, higher plants have evolved versatile mechanisms to cope with different N conditions. In addition, to adaptive changes of the root system architecture (RSA) (Robinson, 1994; Zhang and Forde, 2000), root can regulate its ${\mathrm{NO}}_{3}^{-}$ uptake system to increase N acquiring capacity and utilization efficiency (Crawford and Glass, 1998; Lejay et al., 1999; Glass, 2003). The first step of the ${\mathrm{NO}}_{3}^{-}$ assimilation pathway is ${\mathrm{NO}}_{3}^{-}$ uptake by root epidermal cells. Previous physiological studies have demonstrated that higher plants have developed two high-affinity transport systems (HATS) for the influx of ${\mathrm{NO}}_{3}^{-}$ into roots (Clarkson, 1986; Glass and Siddiqui, 1995; Crawford and Glass, 1998; Daniel-Vedele et al., 1998), including constitutive (cHATS) and ${\mathrm{NO}}_{3}^{-}$-inducible (iHATS) systems. Both HATS systems are able to take up ${\mathrm{NO}}_{3}^{-}$ at low ${\mathrm{NO}}_{3}^{-}$ concentration (<0.25 mM), and display saturable kinetics in a range of 0.2 to 0.5 mM. The cHATS has a relatively higher affinity for ${\mathrm{NO}}_{3}^{-}$ (*K_m_* values of 6–20 μM) and appears to be active even if there was no ${\mathrm{NO}}_{3}^{-}$ in external medium, whereas the iHATS has a relatively lower affinity for ${\mathrm{NO}}_{3}^{-}$ (*K_m_* values of 13–79 μM) and is specifically stimulated by ${\mathrm{NO}}_{3}^{-}$ supply (Forde and Clarkson, 1999). Once the external ${\mathrm{NO}}_{3}^{-}$ concentration exceeds 0.5 mM, the low-affinity system (LATS) often becomes involved in the flux of ${\mathrm{NO}}_{3}^{-}$ into plant roots, and appears to be constitutively expressed in roots. For most plant species, LATS has a linear relationship with the external ${\mathrm{NO}}_{3}^{-}$, and shows no saturation up to 50 mM (Pace and McClure, 1986; Siddiqi et al., 1990; Kronzucker et al., 1995).

To date, four gene families of ${\mathrm{NO}}_{3}^{-}$-transporters have been identified, including nitrate transporter 1/peptide transporter (NPF; formerly named NRT1/PTR), nitrate transporter 2 (NRT2), chloride channel (CLC) and slow anion associated channel homolog (SLAC/SLAH). Among these gene families, functions of ${\mathrm{NO}}_{3}^{-}$-transporters belonging to the NPFs and NRT2s have been widely investigated. The NPF and NRT2 families include 53 and 7 members (Noguero and Lacombe, 2016), respectively, among which six members (i.e., AtNPF6.3/AtNRT1.1, AtNPF4.6/AtNRT1.1, AtNRT2.1, AtNRT2.2, AtNRT2.4, and AtNRT2.5) have been functionally identified to be involved in root ${\mathrm{NO}}_{3}^{-}$ uptake. *AtNPF6.3* was the first gene identified as a low-affinity transporter (Tsay et al., 1993), but it may show a high-affinity at low external ${\mathrm{NO}}_{3}^{-}$ concentration depending upon its phosphorylation state (Ho et al., 2009). In addition, AtNPF6.3 can function as a ${\mathrm{NO}}_{3}^{-}$ sensor regulating root branching to cope with changing N conditions (Remans et al., 2006a; Mounier et al., 2014). *AtNPF4.6* is active only in the low-affinity range and displays a constitutive expression (Huang et al., 1999). *NRT2.1* and *NRT2.2* are located close to each other in genomic regions and both work in the high-affinity range (Orsel et al., 2004; Li et al., 2007; Pii et al., 2016). Moreover, AtNRT2.1 can sense not only the current external ${\mathrm{NO}}_{3}^{-}$ condition, but also the ${\mathrm{NO}}_{3}^{-}$ availability of the plant, finally regulating RSA to capture ${\mathrm{NO}}_{3}^{-}$ adequately (Little et al., 2005; Remans et al., 2006b). Both AtNRT2.4 and AtNRT2.5 participate in root ${\mathrm{NO}}_{3}^{-}$ uptake under N starvation (Kiba et al., 2012; Lezhneva et al., 2014).

Among ${\mathrm{NO}}_{3}^{-}$-transporters, *AtNRT2.1* has been demonstrated to be the major HATS-type gene involving in root ${\mathrm{NO}}_{3}^{-}$ uptake (Li et al., 2007; Lezhneva et al., 2014). The HATS-type gene is particularly crucial for crops to capture adequate ${\mathrm{NO}}_{3}^{-}$ to maintain the growth and yields, because the concentration of ${\mathrm{NO}}_{3}^{-}$ is often low in soils due to that ${\mathrm{NO}}_{3}^{-}$ is hardly retained in soils and can be easily leached into the groundwater (Tian et al., 2016). To date, a number of *NRT2.1*-homologous genes have been cloned and characterized in plant species including *Arabidopsis thaliana* (Pellizzaro et al., 2015), *Chlamydomonas reinhardtii* (Quesada et al., 1994), *Nicotiana plumbaginifolia* (Quesada et al., 1997), *Hordeum vulgare* (Trueman et al., 1996; Vidmar et al., 2000), *Glycine max* (Amarasinghe et al., 1998), *Triticum aestivum* (Zhao et al., 2004), *Oryza sativa* (Cai et al., 2008), *Zea mays* (Plett et al., 2010; Zamboni et al., 2014), *Vitis vinifera* (Pii et al., 2014), and *Chrysanthemum morifolium* (Gu et al., 2016). Cucumber (*Cucumis sativus* L.) is an important fruit vegetable that is sensitive to soil ${\mathrm{NO}}_{3}^{-}$ in the world (Huang et al., 2009). However, little information is available regarding the molecular characterization of high-affinity ${\mathrm{NO}}_{3}^{-}$ transporter in cucumber. Since the cucumber genome sequence has been published (Huang et al., 2009), here we cloned and elucidated the expression, location, and function of cucumber *NRT2.1* gene (*CsNRT2.1*) through the combination of phylogenetic analyses, quantitative real-time polymerase chain reaction (qRT-PCR), green fluorescent protein (GFP) fusion protein localization, and ^15^N stable isotope tracer technique.

## Materials and Methods

### Plant Materials and Growth Conditions

Seeds of cucumber wild-type (WT) (*Cucumis sativus* L. cv. Xintaimici) and transgenic cucumber lines were surface-sterilized in 4% sodium hypochlorite containing 0.02% (v/v) Tween 20 for 5 min, rinsed four times with sterile water and then germinated on moistened filter paper at 28°C for 26 h in darkness. Germinated seeds were sown onto a hydroponic device (Wang et al., 2016) filled with 4.8 L of a modified Yamazaki nutrient solution (Yamazaki, 1982) containing 1 mM CaCl_2_, 1 mM MgSO_4_, 1.5 mM CaCl_2_, 50 μM NaFe-EDTA, 30 μM H_3_BO_3_, 5 μM MnSO_4_, 1 μM ZnSO_4_, 0.5 μM CuSO_4_, and 0.1 μM Na_2_MoO_4_. The pH of the nutrient solution was adjusted to 6.0 with KOH. The solution was supplemented with KNO_3_ as a sole nitrogen source at the concentrations as indicated in each individual experiment. The full N solution (full N) contained 10 mM ${\mathrm{NO}}_{3}^{-}$. For N limiting conditions, ion equilibrium of the solution was ensured by replacing KNO_3_ by K_2_SO_4_. Germinated seedlings were then transferred in a growth chamber with day/night (14/10 h) cycle at 28°C/18°C and 60–80% relative humidity. Light intensity during the day period was 250 μmol m^-2^ s^-1^. All nutrient solutions were completely replaced every day. Cucumber seedlings were sampled at times as indicated in the figures.

### RNA Extraction and qRT-PCR Analysis

Total RNA was extracted from different cucumber tissues using a RNA plant Plus Reagent (Tiangen Biotech, Co., Beijing, China). The quality and concentration of RNA were assessed by gel electrophoresis (2% agarose) and a spectrophotometer (Nanodrop 2000, Thermo Fisher Scientific, Waltham, MA, United States), respectively. The cDNA was synthesized using the PrimeScript^TM^ RT reagent Kit with gDNA Eraser (Perfect Real Time) (TaKaRa, Japan). qRT -PCR was performed using the SYBR^®^ Premix Ex Taq^TM^ (Tli RNaseH Plus) (TaKaRa, Japan) on the QuantStudio^TM^ 6 Flex real-time PCR system (Applied Biosystems, Foster City, CA, United States). The cucumber *Ubiquitin extension protein* (*UBI-ep*) was used as an internal control, and relative amounts of mRNA were calculated using the comparative threshold cycle method. Four biological and three technical replicates were performed for each gene. Specific primers used for qRT-PCR were listed in Supplementary Table S1.

### Cloning of Cucumber Nitrate High-Affinity Transporter (*CsNRT2.1*) cDNA and Sequence Analysis

We used the nucleotide sequence of *AtNRT2.1* (GenBank Accession No. NM_100684.2) as a query, followed by a BLAST search against the Cucumber Genome Database^1^. A 1593-bp PCR fragment containing the complete *CsNRT2.1* coding sequence was amplified from the root DNA (cDNA) with the specific primers listed in Supplementary Table S1. The thermal cycling consisted of a 5-min initial denaturation at 95°C, followed by 30 cycles of denaturation at 95°C for 30 s, annealing at 55°C for 30 s, and elongation at 72°C for 2 min, and a 10-min final extension at 72°C. PCR products were cloned into pMD 19-T vector (TaKaRa, Japan), and subsequently sequenced. The protein sequence alignment was carried out with the DNAMAN software (version 9.0). The phylogenetic tree based on entire amino acid sequence was constructed using the neighbor-joining method by MEGA7 software after ClustalW alignment with 1000 bootstrap trials (Saitou and Nei, 1987). The prediction of transmembrane domains was performed using the TMHMM predictor^2^.

### Subcellular Localization of CsNRT2.1 Proteins

To investigate the subcellular localization of CsNRT2.1, the open reading frame (ORF) of *CsNRT2.1* without stop codons was amplified using the gene-specific primers listed in Supplementary Table S1. The PCR amplification product was cloned into the pCAMBIA super 1300 vector to generate C-terminal fusion construct CsNRT2.1-enhanced green fluorescent protein (EGFP), and CaMV 35S-EGFP was used as a negative control. The recombinant plasmids were transferred into *Agrobacterium tumefaciens* strain GV3101 by electroporation and then transformed into mature leaves of 5-week-old *Nicotiana benthamiana* plants. The tomato bushy stunt virus gene named *p19* was transformed together with the recombinant plasmid to suppress *CsNRT2.1* gene silencing (Grefen et al., 2008). Two days later, EGFP fluorescence was observed at 488 nm by a confocal laser scanning microscope (Fluoview FV1000, Olympus, Japan).

### RNA Interference (RNAi) Construction and *Agrobacterium*-Mediated Cucumber Transformation

For RNAi construction, the vector pFGC1008 was used. Two 157 bp fragments of *CsNRT2.1* were amplified using the specific primers listed in Supplementary Table S1, followed by two double-digests (*AscI/SwaI* and *BamHI/SpeI* sites, respectively). After that, both PCR amplification products were inserted into the vector respectively. The resulting vector was then transferred into *Agrobacterium tumefaciens* strain LBA4404. At last, both *CsNRT2.1*-RNAi recombinant plasmids were transformed into cucumber cultivar “Xintaimici” using the fresh expanding cotyledon disk transformation modified method as previously described (Sui et al., 2012). Briefly, cucumber seeds were broadcasted on MS medium (Murashige and Skoog, 1962). After 3 days germination at 28°C in darkness, the growth points and the upper halves of cotyledons were removed, while other cotyledons were soaked and vacuum infiltrated in the 1/2 MS liquid medium containing *Agrobacterium tumefaciens* that carried the *CsNRT2.1*-RNAi recombinant plasmid (optical density at 600 nm = 0.6–0.8) for 12 min. Then these explants were placed on the MS medium [containing 0.5 mg L^-1^ 6-benzylaminopurine (6-BA) and 1 mg L^-1^ abscisic acid (ABA)] at 28°C for 3 days in darkness. After that, the explants were transformed into the MS medium containing 0.5 mg L^-1^ 6-BA, 1 mg L^-1^ ABA, 25 mg L^-1^ Kanamycin, and 500 mg L^-1^ carbenicillin, and then cultivated for 2–3 weeks at 28°C with day/night (12/12 h) cycle under 250 μmol m^-2^ s^-1^ photon flux density. The shoots differentiated from the explants were transformed to the MS medium containing 100 mg L^-1^ kanamycin and 200 mg L^-1^ carbenicillin for root initiation and shoot growth.

### Root ^15^${\mathrm{NO}}_{3}^{-}$ Influx and Kinetics of ^15^${\mathrm{NO}}_{3}^{-}$ Influx

Root influx and net uptake of ${\mathrm{NO}}_{3}^{-}$ were assayed by ^15^N labeling as described in Delhon et al. (1995). WT and *CsNRT2.1*-RNAi lines were grown on the hydroponic device mentioned above. Seedlings were first grown in the full (10 mM ${\mathrm{NO}}_{3}^{-}$) N nutrient solution for 20 days, and then transferred to N-free solution for 5 days before **^15^${\mathrm{NO}}_{3}^{-}$** labeling. Prior to measuring **^15^${\mathrm{NO}}_{3}^{-}$** influx, seedlings were transferred to 0.1 mM CaSO_4_ for 1 min, and then to a complete nutrient solution containing **^15^${\mathrm{NO}}_{3}^{-}$** (atom% ^15^N: 99%) at the indicated concentrations for 10 min. Roots were washed again in 0.1 mM CaSO_4_ for 1 min and separated from shoots after **^15^${\mathrm{NO}}_{3}^{-}$** labeling. The roots were dried at 85°C for 48 h and then crushed in a hammer mill immediately. Total N and atom %^15^N were measured using a continuous flow isotope ratio mass spectrometer (ANCA-MS, PDZ Europa, Crewe, United Kingdom). Influx of **^15^${\mathrm{NO}}_{3}^{-}$** was calculated from the total N and ^15^N content and expressed in μmol h^-1^ g^-1^ dry weight. To obtain the kinetics of **^15^${\mathrm{NO}}_{3}^{-}$** influx, data were calculated based on Michaelis–Menten equation to obtain *V_max_* and *K_m_* estimates.

### Measurement of Root Morphology

Cucumber root systems were scanned at 300 dpi using a special scanner (Expression 4990, Epson, Long Beach, CA, United States). Root-related growth parameters (**Table 1**) were determined after analysis of scanned images with a computer image-analysis software (Win RHIZO, Regent Instruments, Inc., Canada) and ImageJ software (V1.50b) (Abràmoff et al., 2004).

**Table 1:** Root morphological characteristics quantified in this study.

Abbreviation	Description
PRS	Primary root length
TRS	Total root length
1st LRS	Sum of path length of the first-order LRs (emerging form the PR)
1st order LR no.	Number of the first-order LRs
1st LRP	Mean LR path length of the first-order LRs
2nd LRS	Sum of path length of the second-order LRs (emerging from first-order LRs)
2nd order LR no.	Number of the second-order LRs
2nd LRP	Mean LR path length of the second-order LRs

### Statistical Analysis

Statistical analysis was performed by the one-way analysis of variance (ANOVA) using SPSS software version 22.0 (SPSS, Inc., Chicago, IL, United States), and Tukey’s honestly significant difference (HSD) *post hoc* test was employed to detect differences between WT and transgenic cucumber lines.

## Results

### Isolation and Sequence Analysis of *CsNRT2.1*

**FIGURE 1:**
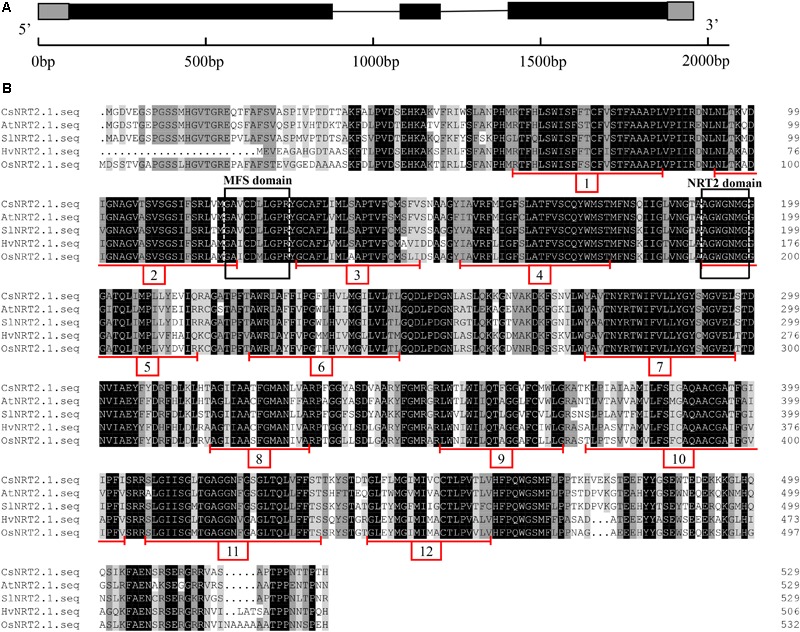
The structure of *CsNRT2.1* **(A)**, alignment of NRT2.1 amino acid sequence from *Cucumis sativus, Arabidopsis thaliana, Solanum lycopersicum, Hordeum vulgare*, and *Oryza sativa* **(B)**. Alignment was performed with DNAman. Exons, introns, and upstream/downstream in **(A)** were represented by black boxes, black lines, and gray boxes, respectively. The red lines indicated the transmembrane domain in **(B)**.

The putative cDNA sequence encoding *CsNRT2.1* (GenBank Accession No. MH213459) was isolated from cucumber roots, and the full-length was 1909 bp. It contained a 1593 bp ORF (Supplementary Figure S1) encoding 530 amino acids (Supplementary Data) with a predicted molecular mass of 57.71 kDa. The structure analysis showed that CsNRT2.1 had a 45 bp 5′ untranslated region (UTR), a 33 bp 3′ UTR, three exons and two introns (**Figure 1A**). The protein sequence alignment predicted that CsNRT2.1 had 12 transmembrane domains (TMs) in the major facilitator superfamily (MFS) (**Figure 1B** and Supplementary Figure S2). In addition, a MFS-specific domain (G-X3-D-X2-G-X-R) was identified between the TMs 2 and 3, and a nitrate/nitrite transporters family motif (A-G-W/L-G-N-M-G) was observed in the TM 5 (**Figure 1B**), respectively.

**FIGURE 2:**
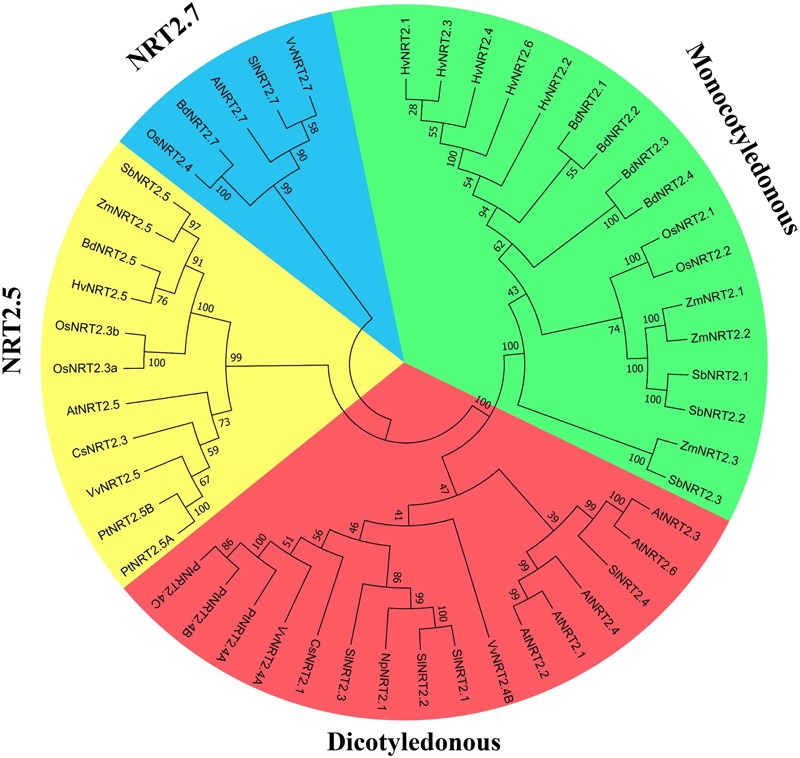
Phylogenetic analysis of NRT2 proteins. The amino acid sequences were aligned using ClustalW software and the phylogeny constructed using the neighbor-joining method with 1000 replicates through MEGA7 software. Database accession numbers of the amino acid sequences used are: *Cucumis sativus* CsNRT2.1 (MH213459), CsNRT2.3 (NP_001295862); *Arabidopsis thaliana* AtNRT2.1 (NP_172288), AtNRT2.2 (NP_172289), AtNRT2.3 (NP_200886), AtNRT2.4 (NP_200885), AtNRT2.5 (NP_172754), AtNRT2.6 (NP_190092), AtNRT2.7 (NP_196961); *Populus trichocarpa* PtNRT2.4A (POPTR_0009s01410.1), PtNRT2.4B (POPTR_0143s00200.1), PtNRT2.4C (POPTR_0009s01420.1), PtNRT2.5A (POPTR_0015s09290.1), PtNRT2.5B (POPTR_0015s09310.1); *Vitis vinifera* VvNRT2.4A (VIT_06s0061g00320), VvNRT2.4B (VIT_08s0040g01500), VvNRT2.5 (VIT_01s0127g00070), VvNRT2.7 (VIT_14s0066g00850); *Sorghum bicolor* SbNRT2.1 (Sb04g001000.1), SbNRT2.2 (Sb04g000990.1), SbNRT2.3 (Sb04g000970.1), SbNRT2.5 (Sb03g032310.1); *Solanum lycopersicum* SlNRT2.1 (AAF00053), SlNRT2.2 (NP_001266263), SlNRT2.3 (NP_001234127), SlNRT2.4 (XP_004240585), SlNRT2.7 (XP_004233327); *Glycine max* CmNRT2.1 (NP_001236444), CmNRT2.4 (XP_003539195); *Hordeum vulgare* HvNRT2.1 (AAC49531), HvNRT2.2 (AAC49532), HvNRT2.3 (AAD28363), HvNRT2.4 (AAD28364), HvNRT2.5 (ABG20828), HvNRT2.6 (ABG20829); *Zea mays* ZmNRT2.1 (AAN05088), ZmNRT2.2 (AAN05088), ZmNRT2.3 (XP_008645163), ZmNRT2.5 (XP_008656795); *Brachypodium distachyon* BdNRT2.1 (XP_003572550.1), BdNRT2.2 (XP_003572454.1), BdNRT2.3 (XP_003572590.2), BdNRT2.4 (XP_003570801.1), BdNRT2.5 (XP_003569637.1), BdNRT2.7 (XP_003566766.2); *Oryza sativa* OsNRT2.1 (AB008519), OsNRT2.2 (AK109733), OsNRT2.3a (AK109776), OsNRT2.3b (AK072215), OsNRT2.4 (LOC_Os01g3672); *Vitis vinifera* VvNRT2.1 (XP_002277127); *Nicotiana plumbaginifolia* NpNRT2.1 (CAA69387).

The unrooted phylogenetic tree (**Figure 2**) showed that NRT2 proteins could be clustered into four groups that included dicotyledonous and monocotyledonous plants, and clades NRT2.5 and NRT2.7. CsNRT2.1 showed a high degree of homology to genes in dicotyledonous plants and particularly to PtNRT2.4A, PtNRT2.4B, PtNRT2.4C, and VvNRT2.4A.

### Subcellular Localization of CsNRT2.1

**FIGURE 3:**
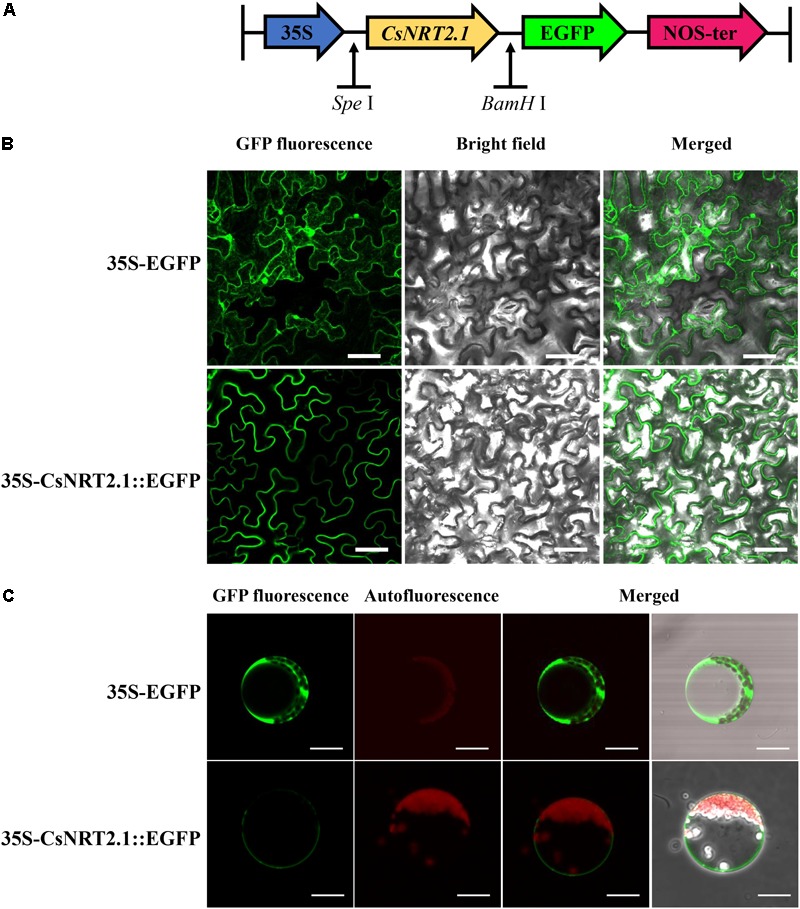
Subcellular localization of the CsNRT2.1. **(A)** The schematic diagram of the 35S-CsNRT2.1::EGFP construct. **(B)** Transient expression of the 35S-CsNRT2.1::EGFP fusion protein in tobacco leaf epidermal cells. Bar = 50 μm. **(C)** Subcellular localization of the 35S-CsNRT2.1::EGFP fusion protein in Arabidopsis mesophyll protoplasts. Bar = 10 μm.

To determine the subcellular localization of CsNRT2.1, 35S-CsNRT2.1::EGFP fusion constructs (**Figure 3A**) and the positive control 35S-EGFP were transiently transformed into leaf cells of *Nicotiana benthamiana* using the agroinfiltration The results showed that CsNRT2.1::EGFP was only expressed in the plasma membrane, whereas the 35S-EGFP was detected not only in the plasma membrane, but also in the cytoplasm and nucleus (**Figure 3B**). Similar results were also obtained in Arabidopsis mesophyll protoplasts (**Figure 3C**).

### Expression Pattern of *CsNRT2.1* in Cucumber Plants

**FIGURE 4:**
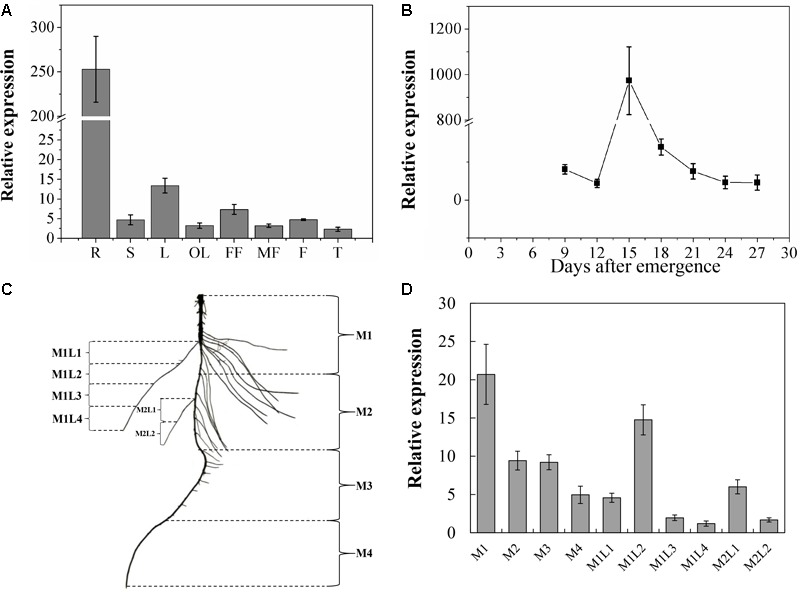
Quantitative real-time polymerase chain reaction (qRT-PCR) analysis of the *CsNRT2.1* spatiotemporal expression. **(A)** *CsNRT2.1* expression was analyzed on plants cultivated in full N (10 mM ${\mathrm{NO}}_{3}^{-}$) under hydroponic condition for 45 days. R, root; S, stem; L, leaf; OL, old leaf; FF, female flower; MF, male flower; F, fruit; T, tendril. **(B)** Expression level of the *CsNRT2.1* in roots throughout the seedling stage. Cucumber seedlings were cultivated in full N (10 mM ${\mathrm{NO}}_{3}^{-}$) in hydroponic condition for 27 days. **(C)** Schematic model of the areas of *CsNRT2.1* gene expression in cucumber roots. Plants were grown on hydroponic condition for 15 days. **(D)** *CsNRT2.1* expression in different areas of cucumber root. UBI in cucumber was used as an internal control, and error bars represent standard error (SE) of three technical replicates of five biological replicates.

The spatiotemporal expression analysis showed that although CsNRT2.1 was expressed in all examined plant tissues, the relative expression level was much higher in roots than in other plant tissues (**Figure 4A**). Since the root was the major tissue expressing *CsNRT2.1*, we subsequently analyzed the temporal (**Figure 4B**) and spatial (**Figures 4C,D**) expression patterns of *CsNRT2.1* in the root system of cucumber seedlings grown in full N (10 mM ${\mathrm{NO}}_{3}^{-}$). In the temporal pattern, the relative expression level of *CsNRT2.1* increased rapidly and reached a maximum on day 15, and then decreased gradually to reach a relatively constant level by day 21 (**Figure 4B**). In the spatial pattern, *CsNRT2.1* was expressed in all root sections (for more details regarding root sections see **Figure 4C**) and mainly in the older portions of both primary and lateral roots (compare M1 versus M2, M3 and M4, compare M1L1 and M1L2 versus M1L3 and M1L4, and compare M2L1 versus M2L2; **Figure 4D**).

### Expression Profiles of *CsNRT2.1* in Response to N Availability

**FIGURE 5:**
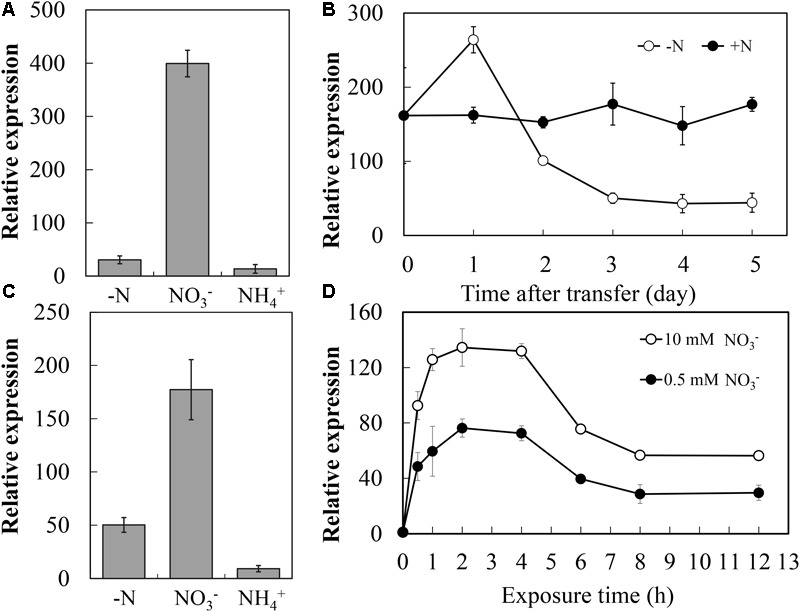
Expression of the *CsNRT2.1* in young seedlings in respond to N availability. **(A)** Relative expression of *CsNRT2.1* in the root and root of seedlings grown under different N conditions. WT cucumber seedlings were grown in 10 mM ${\mathrm{NO}}_{3}^{-}$ for 10 days and incubated in 10 mM KNO_3_ (${\mathrm{NO}}_{3}^{-}$), 5 mM ${\mathrm{NH}}_{4}^{+}$ (${\mathrm{NH}}_{4}^{+}$), or no N (–N) for 3 days. **(B)** Expression levels of *CsNRT2.1* in the root during N starvation period. WT cucumber seedlings grown on full N (10 mM ${\mathrm{NO}}_{3}^{-}$) hydroponic conditions for 10 days were transferred to hydroponic conditions containing 10 mM ${\mathrm{NO}}_{3}^{-}$ (+N), or 0 mM ${\mathrm{NO}}_{3}^{-}$ (–N) and harvested at the indicated times. **(C)** Effect of N resupply on the expression of *CsNRT2.1*. WT seedlings were germinated and grown on full N (10 mM ${\mathrm{NO}}_{3}^{-}$) in hydroponic condition for 10 days and thereafter transferred to 0 mM ${\mathrm{NO}}_{3}^{-}$, and then resupplied with either 10 mM ${\mathrm{NO}}_{3}^{-}$ (${\mathrm{NO}}_{3}^{-}$) or 5 mM (${\mathrm{NH}}_{4}^{+}$) 2 succinate (${\mathrm{NH}}_{4}^{+}$) for 2 h compared to a control (–N) without resupply. **(D)** Expression levels of *CsNRT2.1* in N starved root after N induction. Wild-type (WT) seedlings grown on full N (10 mM ${\mathrm{NO}}_{3}^{-}$) for 10 days, N starvation for 5 days and then exposure to 10 mM ${\mathrm{NO}}_{3}^{-}$. UBI in cucumber was used as an internal control. ND indicated not detected in **(A,C)**. Error bars represent SE of three technical replicates of five biological replicates.

To analyze the expression of *CsNRT2.1* in response to different N sources, young cucumber seedlings were grown in full N (10 mM ${\mathrm{NO}}_{3}^{-}$) for 2 weeks and then transferred to different N sources (10 mM ${\mathrm{NO}}_{3}^{-}$ or 5 mM ${\mathrm{NH}}_{4}^{+}$) or to nutrient solution without N for 3 days. Compared with the ${\mathrm{NO}}_{3}^{-}$-replete control where *CsNRT2.1* was expressed at high levels, *CsNRT2.1* expression in roots was decreased by 71.6 and 94.8% under N starvation and 5 mM ${\mathrm{NH}}_{4}^{+}$ conditions, respectively (**Figure 5A**).

Given the significant decreased expression of *CsNRT2.1* in N-depleted roots, we followed the time-course pattern of *CsNRT2.1* expression during N starvation. To do so, 10-day-old seedlings were transferred from full N nutrient solution to either the N-free or full N nutrient solution, and roots samples were taken on days 0, 1, 2, 3, 4, and 5 after transfer to measure gene expression (**Figure 5B**). After transfer to N-free nutrient solution, *CsNRT2.1* expression increased rapidly to a maximum on day 1, but decreased gradually to a relatively constant and lower level at day 0. Under the full N condition, however, *CsNRT2.1* expression throughout maintained a relatively stable and higher level, indicating the essential role of ${\mathrm{NO}}_{3}^{-}$ in maintaining high *CsNRT2.1* expression.

To demonstrate the inducing role of ${\mathrm{NO}}_{3}^{-}$ in *CsNRT2.1* expression, 10-day-old seedlings grown in the full N nutrient solution were first transferred to the N-free nutrient solution for 5 days and then to different N sources (10 mM ${\mathrm{NO}}_{3}^{-}$ or 5 mM ${\mathrm{NH}}_{4}^{+}$) or to the nutrient solution without N (-N) for 2 h. The results showed that *CsNRT2.1* expression was mainly induced by ${\mathrm{NO}}_{3}^{-}$, but was repressed by ${\mathrm{NH}}_{4}^{+}$ (**Figure 5C**). Given the ${\mathrm{NO}}_{3}^{-}$ could induce the CsNRT2.1, we analyzed the detailed expression profile of CsNRT2.1 within a short time. The time-course pattern showed that under the ${\mathrm{NO}}_{3}^{-}$-induced condition, *CsNRT2.1* expression increased rapidly and reached a maximum at 2 h after induction, and then decreased gradually to reach a relatively constant level at 8 h after induction (**Figure 5D**).

### Construction of *CsNRT2.1*-RNAi Lines

**FIGURE 6:**
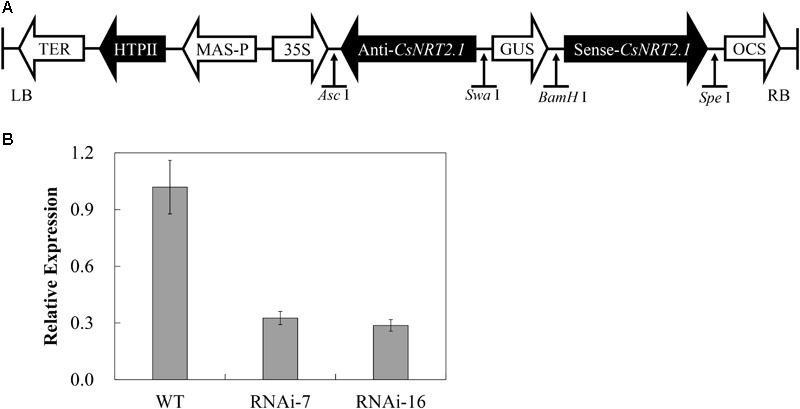
Construction of *CsNRT2.1*-RNAi T2 generation cucumber lines. **(A)** The schematic diagram of *CsNRT2.1*-RNAi construct. **(B)** The relative expression analyses of *CsNRT2.*1 in WT, *CsNRT2.1*-RNAi lines grown on 0.5 ${\mathrm{NO}}_{3}^{-}$ hydroponic conditions by qRT-PCR. UBI in cucumber was used as an internal control. Error bars represent SE of three technical replicates of five biological replicates.

To investigate the function of *CsNRT2.1*, a double-strand RNAi vector containing the *CsNRT2.1*-specific sequence was constructed under control of the 35S promoter (**Figure 6A**). Then, the vector was introduced into cucumber cotyledons by agroinfiltration, and 25 transgenic plants were obtained. The qRT-PCR results showed that the expression level of *CsNRT2.1* was significantly decreased by 68.0% in RNAi-7 and 71.8% in RNAi-16, respectively, when compared with WT (**Figure 6B**). Therefore, these two lines were used for further studies.

### CsNRT2.1 Is a ${\mathrm{NO}}_{3}^{-}$-Induced High-Affinity Nitrate Transporter

**FIGURE 7:**
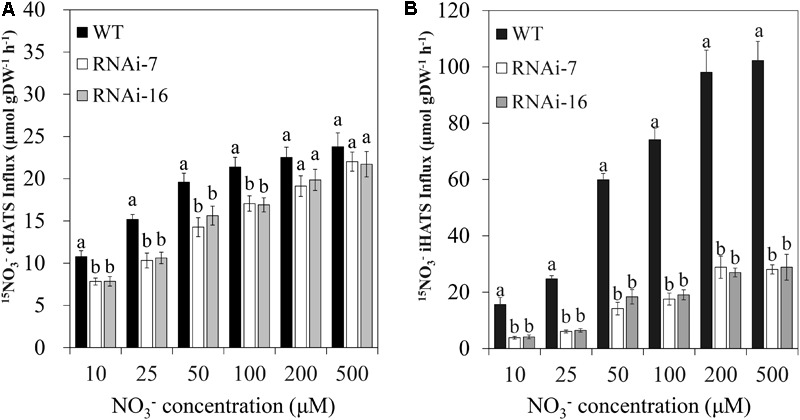
Root ^15^${\mathrm{NO}}_{3}^{-}$ influx in WT, RNAi-7, and RNAi-16. WT and RNAi plants grown in 10 mM KNO_3_ for 25 days and then deprived of N for 5 days. **(A)** ^15^${\mathrm{NO}}_{3}^{-}$ influx was measured immediately from solutions containing various ^15^${\mathrm{NO}}_{3}^{-}$ concentrations. **(B)** N-starved plant roots were then exposed to 1 mM KNO_3_ for 6 h prior to measuring ^15^${\mathrm{NO}}_{3}^{-}$ influx at various ^15^${\mathrm{NO}}_{3}^{-}$ concentrations. The values are means ± standard error of five biological replicates. Different letters over the bars denote significance at *P* < 0.05 by Tukey’s HSD-test. gDW, gram dry weight.

To determine the CsNRT2.1 function in ${\mathrm{NO}}_{3}^{-}$ uptake by roots, we measured both cHATS and iHATS in cucumber roots. Cucumber seedlings were grown in full N (10 mM ${\mathrm{NO}}_{3}^{-}$) for 25 days and then transferred to the free N nutrient solution for 5 days to deinduce the ${\mathrm{NO}}_{3}^{-}$ transport. After that, these N-starved seedlings were exposed to various concentrations of **^15^${\mathrm{NO}}_{3}^{-}$** (from 10 to 500 μM), and the influx measured was the cHATS. Alternatively, N-starved seedlings were treated with 1 mM KNO_3_ for 6 h and then exposed to **^15^${\mathrm{NO}}_{3}^{-}$**, and the influx measured was the combination of cHATS and iHATS (ciHATS). The iHATS was calculate by subtracting the cHATS from the ciHATS. The results showed that the cHATS activity was significantly lower in transgenic lines (RNAi-7 and RNAi-16) than in the WT at relatively lower (10–100 μM) **^15^${\mathrm{NO}}_{3}^{-}$** concentrations (**Figure 7A**). However, no significant difference was found at relatively higher (200 and 500 μM) **^15^${\mathrm{NO}}_{3}^{-}$** concentrations (**Figure 7A**). By contrast, the iHATS activity was significantly lower in transgenic lines than in the WT at all tested **^15^${\mathrm{NO}}_{3}^{-}$** concentrations (**Figure 7B**). The results from Michaelis–Menten equation showed that the *V_max_* was significantly decreased by 65.1% in RNAi-7 and 62.8% in RNAi-16 lines, respectively, when compared to the WT (**Table 2**). However, no significant difference was found in the *K_m_*.

**Table 2:** *V_max_* (μmol gDW^-1^ h^-1^) and *K_m_* (μM) values for ^15^${\mathrm{NO}}_{3}^{-}$ influx in WT, RNAi-7, and RNAi-16.

	WT	RNAi-7	RNAi-16
*V_max_*	115 ± 9.47a	40.2 ± 3.27b	42.8 ± 2.89b
*K_m_*	66.7 ± 5.77a	72.4 ± 4.68a	73.1 ± 7.54a

*Plants were grown in 10 mM KNO_*3*_ for 25 days and then deprived of N for 5 days. Roots were then exposed to 1 mM KNO_*3*_ for 6 h prior to measuring **^*15*^${\mathrm{NO}}_{3}^{-}$** influx at various **^*15*^${\mathrm{NO}}_{3}^{-}$** concentrations. Values shown are means (*n* = 30) ± standard error. Different letters between the three lines denote significance at *P* < 0.05 by Tukey’s HSD-test.*

In addition to cHATS and iHATS, LATS was also measured through exposing N-starved seedlings that were treated with 1 mM KNO_3_ for 6 h to different concentrations of **^15^${\mathrm{NO}}_{3}^{-}$** (1, 5, 10, and 20 mM). According to the measured influx, no significant difference in the LATS activity was found between transgenic lines and the WT (Supplementary Figure S3).

### Knock-Down of *CsNRT2.1* Strongly Affects Root System Architecture Under Low ${\mathrm{NO}}_{3}^{-}$ Condition

**FIGURE 8:**
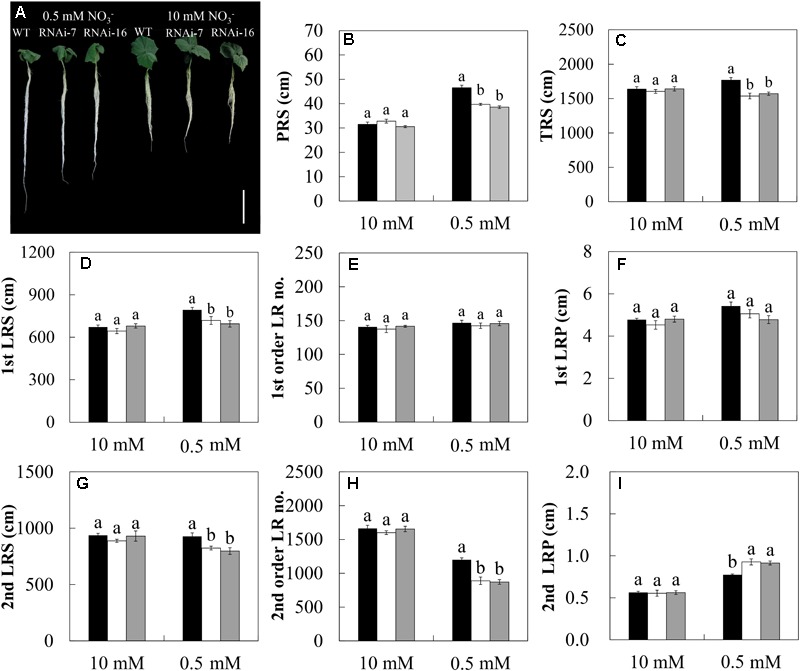
The root morphological characteristics of cucumber seedlings in WT (black bars), RNAi-7 (white bars), and RNAi-16 (gray bars) lines. Cucumber seedlings were grown in 10 or 0.5 mM ${\mathrm{NO}}_{3}^{-}$ conditions for 15 days. **(A)** Morphological changes of cucumber seedlings. The root parameters of PRS **(B)**, TRS **(C)**, 1st LRS **(D)**, 1st order LR no. **(E)**, 1st LRP **(F)**, 2nd LRS **(G)**, 2nd order LR no. **(H)**, and 2nd LRP **(I)** are described in **Table 1**. Different letters over the bars denote significance at *P* < 0.05 by Tukey’s HSD-test between the different lines. Values shown are means ± standard error of five biological replicates. Bar = 10 cm.

Besides the HATS, the RSA is another important factor for plants to respond different N conditions. Since the expression level of *CsNRT2.1* in roots was considerably high on day 15 after emergence (**Figure 3B**), germinated seeds of WT and two RNAi lines were grown in 10 or 0.5 mM ${\mathrm{NO}}_{3}^{-}$ for 15 days, and the RSA was measured based on various root parameters (**Table 1**). In general, under low ${\mathrm{NO}}_{3}^{-}$ condition (0.5 mM), the PRS, TRS, 1st LRS, 2nd LRS, and 2nd order LR no. were reduced, while the 2nd LRP was increased by both RNAi-7 and RNAi-16 compared to the WT (**Figure 8**). Under high ${\mathrm{NO}}_{3}^{-}$ condition (10 mM), however, no significant difference was found in all measured root parameters between the transgenic lines and the WT under low ${\mathrm{NO}}_{3}^{-}$ condition (0.5 mM).

## Discussion

To date, two NRT families (i.e., NRT1 and NRT2) have been identified to be involved in ${\mathrm{NO}}_{3}^{-}$ uptake by roots, and the HATS-type NRT2 is more important for plants grown in soils with relatively low and changeable ${\mathrm{NO}}_{3}^{-}$. Although several *NRT2* genes have been isolated in the model plant Arabidopsis, little information is available regarding cucumber, an important vegetable crop in the world (FAO, 2017). In this study, we isolated a *NRT2* gene from cucumber named *CsNRT2.1*, and investigated its spatiotemporal expression and function. Our data clearly showed that CsNRT2.1 had all typical features of the HATS-type NRT2 (Forde, 2000), including 12 TMs in the MFS (Saier et al., 1999), the MFS-conserved motif (G-X3-D-X2-G-X-R), and the nitrate/nitrite transporters family-conserved motif (A-G-W/L-G-N-M-G).

In consistent with the accumulation pattern of *NRT2.1* mRNA in Arabidopsis (Zhuo et al., 1999; Nazoa et al., 2003), *CsNRT2.1* was predominantly expressed in roots and especially in mature portions (**Figure 3A**). However, in roots the *CsNRT2.1* expression showed a first increasing and then decreasing trend (**Figure 3B**), which was consistent with the *ZmNRT2.1* expression profile across the life-cycle (Garnett et al., 2013). This is probably due to the fact that Cs*NRT2.1* expression was modulated by plant N demand. It has long been suggested that the plant N demand decreases during the transition from vegetative growth to reproductive growth (York et al., 2016).

Previous studies showed that *NRT2.1* expression could be induced in higher plant species by nitrate in a broad range of concentrations (from 0.2 mM in Arabidopsis to 25 mM in non-heading Chinese cabbage; Amarasinghe et al., 1998; Zhuo et al., 1999; Tong et al., 2005; Araki and Hasegawa, 2006; Pellizzaro et al., 2015). In this study, *CsNRT2.1* expression could maintain a relatively constant and high level when seedlings were grown in 10 mM ${\mathrm{NO}}_{3}^{-}$ (**Figure 5B**). In addition, when N-starved plants were exposed to ${\mathrm{NO}}_{3}^{-}$, CsNRT2.1 expression was higher under full N (10 mM) than under N-limited (0.5 mM) conditions (**Figure 5D**). However, Gu et al. (2016) reported that the expression of *CmNRT2.1* in chrysanthemum was threefold higher under N-limited (0.5 mM) than under full N (5 mM) conditions. The reverse expression profile suggested that different plant species showed different NRT2.1 responses to ${\mathrm{NO}}_{3}^{-}$.

It noted that once exposed to ${\mathrm{NO}}_{3}^{-}$, *CsNRT2.1* expression in N-starved plants showed first increasing and then decreasing trends (**Figure 5D**). This result suggested that *CsNRT2.1* was feedback-repressible by N metabolites in plants. It has been extensively demonstrated that the ${\mathrm{NO}}_{3}^{-}$ influx and the NRT expression can be inhibited by N metabolites because of high systemic N status (Alvarez et al., 2012). More importantly, for both N-sufficient and N-starved plants, exposure to ${\mathrm{NH}}_{4}^{+}$ suppressed *CsNRT2.1* expression (**Figures 5A,C**), further indicating the feedback inhibition by N metabolites. In addition to N metabolites, photosynthate (e.g., sucrose) may also influence *NRT2.1* expression. This has been widely verified in several plant species, such as Arabidopsis (Little et al., 2005), soybean (Delhon et al., 1995), tomato (Tucker et al., 2004), and *Medicago truncatula* (Pellizzaro et al., 2015).

In Arabidopsis, *AtNRT2.1* was the major HAT-type ${\mathrm{NO}}_{3}^{-}$ transporter in response to low ${\mathrm{NO}}_{3}^{-}$ condition, because the iHATS activity of the mutant disrupted in *NRT2.1* could be reduced by up to 72% (Li et al., 2007). Based on our results, it seems that the expression profile of *CsNRT2.1* was similar to that of *AtNRT2.1*, which operated the function of ${\mathrm{NO}}_{3}^{-}$ uptake under low ${\mathrm{NO}}_{3}^{-}$ condition. Firstly, for both RNAi-7 and RNAi-16, the cHATS influx was significantly (*P* < 0.05) reduced only at relatively low ${\mathrm{NO}}_{3}^{-}$ concentrations (e.g., 10–100 μM; **Figure 7A**), while the iHATS influx was reduced at all tested ${\mathrm{NO}}_{3}^{-}$ concentrations (e.g., 10–500 μM; **Figure 7B**). Secondly, under the same low ${\mathrm{NO}}_{3}^{-}$ condition, the reduction of iHATS influx was generally much higher than that of cHATS influx in transgenic lines (**Figures 7A,B**). Finally, *V_max_* values were also significantly reduced in transgenic lines (**Table 2**). Despite all of this, there were still some differences in the NRT2.1 expression between cucumber and Arabidopsis. For Arabidopsis, the reduction of cHATS influx only occurred in the mutant disrupted in both *AtNRT2.1* and *AtNRT2.2* (Li et al., 2007). However, for cucumber, knock-down of *CsNRT2.1* alone could significantly reduce the cHATS influx (**Figure 7A**).

In addition to the regulation of the HATS, plants modulated the spatial arrangement of RSA to cope with fluctuating ${\mathrm{NO}}_{3}^{-}$ availabilities. In Arabidopsis, RSA can be regulated by both external ${\mathrm{NO}}_{3}^{-}$ concentration and the endogenous N status of the plant (Krapp et al., 2014). A lot of studies have revealed that the influence of external ${\mathrm{NO}}_{3}^{-}$ concentration on lateral root elongation strongly depends on the acceleration of the meristematic activity of mature LR tips (Zhang et al., 1999; Zhang and Forde, 2000; Remans et al., 2006b). Therefore, it seems that sufficient ${\mathrm{NO}}_{3}^{-}$ supply is an indispensable factor for root elongation. In the present study, however, for the WT plants, the PRS, 1st LRS, 1st LRP, and 2nd LRP were significantly higher under N-limitation than under full N condition (**Figures 8A,B,D,F,I**). This result indicates that under N limitation, the modification of RSA depends on the degree to which the plants are stressed. Generally, root length is increased under mild N limitation but is decreased under severe N limitation (Gruber et al., 2013). Previous studies showed that the external ${\mathrm{NO}}_{3}^{-}$ supply mainly affected the LR length rather than LR number (Zhang et al., 1999; Mounier et al., 2014). In this study, however, a significant increase in 2nd order LR no. in WT was observed under full N condition compared to the N limitation (**Figure 8H**). This result was also verified by a recent study that applying uniformly ${\mathrm{NO}}_{3}^{-}$ to the whole root system stimulated an apparent increase in LR number (Vidal et al., 2013).

Knock-down of *CsNRT2.1* markedly altered the RSA response to ${\mathrm{NO}}_{3}^{-}$ limitation by increasing the PRS, TRS, 1st LRS, 2nd LRS, and 2nd order LR no. and decreasing the 2nd LRP (compare RNAi lines versus WT; **Figures 8A,B,C,D,G,I**), indicating the important role played by *CsNRT2.1* in regulating root growth under low ${\mathrm{NO}}_{3}^{-}$ condition. Similar trends were also found in *atnrt2.1-1* mutant (deleted for both *NRT2.1* and *NRT2.2*) of Arabidopsis grown under ${\mathrm{NO}}_{3}^{-}$ limitation (Remans et al., 2006b). Interestingly, however, a significant decrease of PRS was observed in our RNAi lines (**Figure 8A**) but not in *atnrt2.1* mutant (Remans et al., 2006b). Since the primary root growth is generally determined by water supply rather than ${\mathrm{NO}}_{3}^{-}$ supply (Chapman et al., 2011), CsNRT2.1 might regulate the primary root growth through altering the root hydraulic conductivity. This could be supported by a recent study which showed a significant reduction of root hydraulic conductivity in *atnrt2.1-1* mutant (Li et al., 2016). On the other hand, the decreased PRS in our RNAi lines (**Figure 8A**) might also be associated with the decreased auxin concentration at the root tip (Vidal et al., 2010). However, the interaction between the auxin-mediated signaling pathway and the regulation of CsNRT2.1 needs to be examined further.

In summary, CsNRT2.1 is a high affinity nitrate transporter expressed mainly in cucumber roots. Similar to the *NRT2.1* in some species such as *Arabidopsis thaliana* and *Nicotiana plumbaginifolia, CsNRT2.1* is involved in nitrate uptake at low external nitrate concentration. However, unlike most previous studies, which generally showed that plants disrupted in *NRT2.1* had a reduced iHATS, this study demonstrated that the disruption of *CsNRT2.1* decreased not only the iHATS but also the cHATS. In addition, under low nitrate conditions, the *CsNRT2.1* influenced RSA mainly through reducing the root length and lateral root numbers. It noted that the reduced main root length in the *CsNRT2.1* knock-down plants has not been reported in previous studies.

## Author Contributions

YL, YT, LG, and WZ conceived and designed the experiments. YL, JL, YY, and WL performed the experiments. YL and YT analyzed the data and wrote the paper.

## Conflict of Interest Statement

The authors declare that the research was conducted in the absence of any commercial or financial relationships that could be construed as a potential conflict of interest. The reviewer YP and handling Editor declared their shared affiliation.

## Footnotes

1 http://cucurbitgenomics.org/blast

2 http://www.cbs.dtu.dk/services/TMHMM
