# Supplementary material for: Knock-Down of CsNRT2.1, a Cucumber Nitrate Transporter, Reduces Nitrate Uptake, Root length, and Lateral Root Number at Low External Nitrate Concentration
Source: Front Plant Sci. 2018 Jun 1;9:722. doi: 10.3389/fpls.2018.00722 (PMC5992502; doi:10.3389/fpls.2018.00722)
Supplement: Supplementary file 1 [file Data_Sheet_1.PDF]

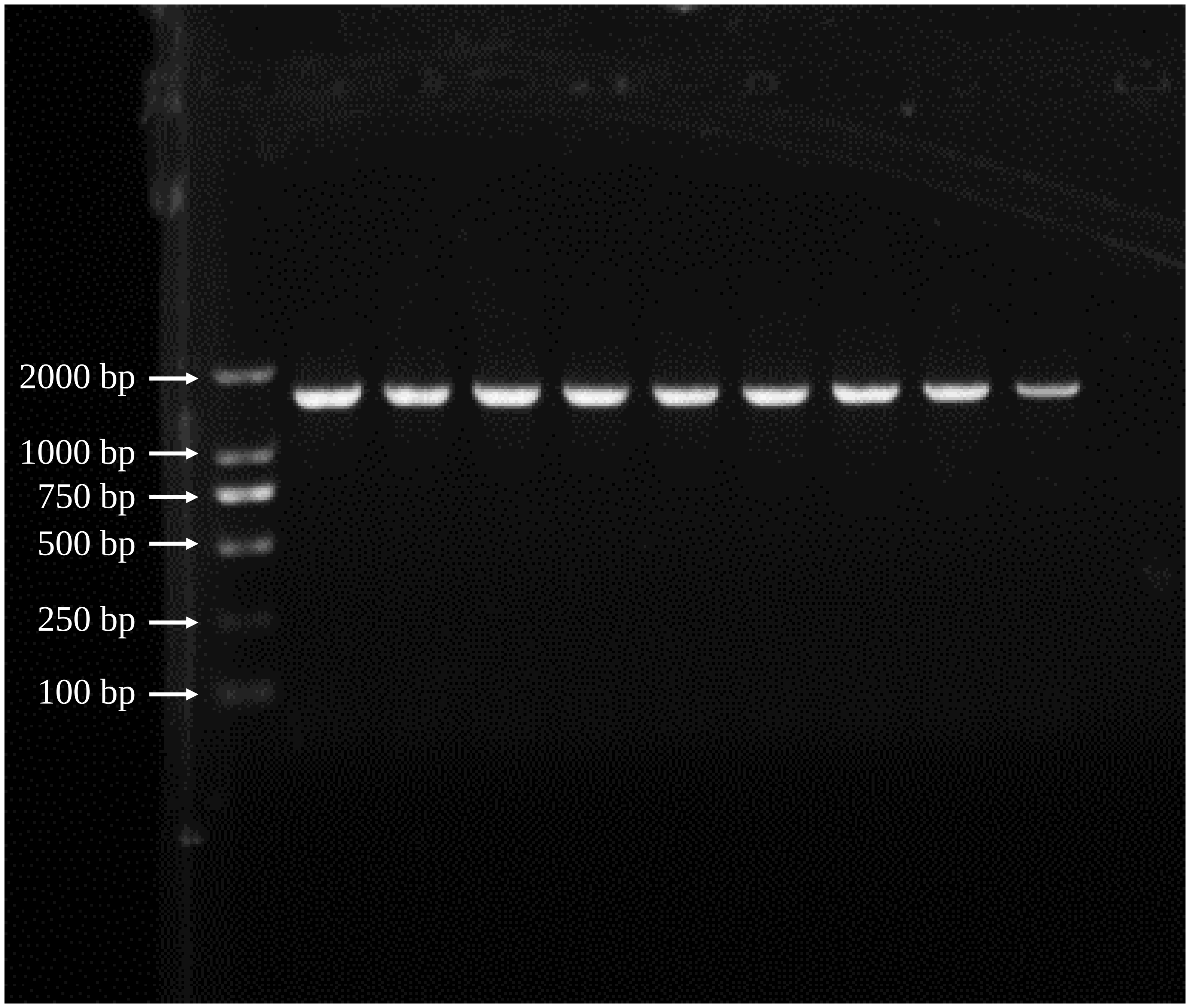

Supplementary Figure S1. Cloning of the CsNRT2.1

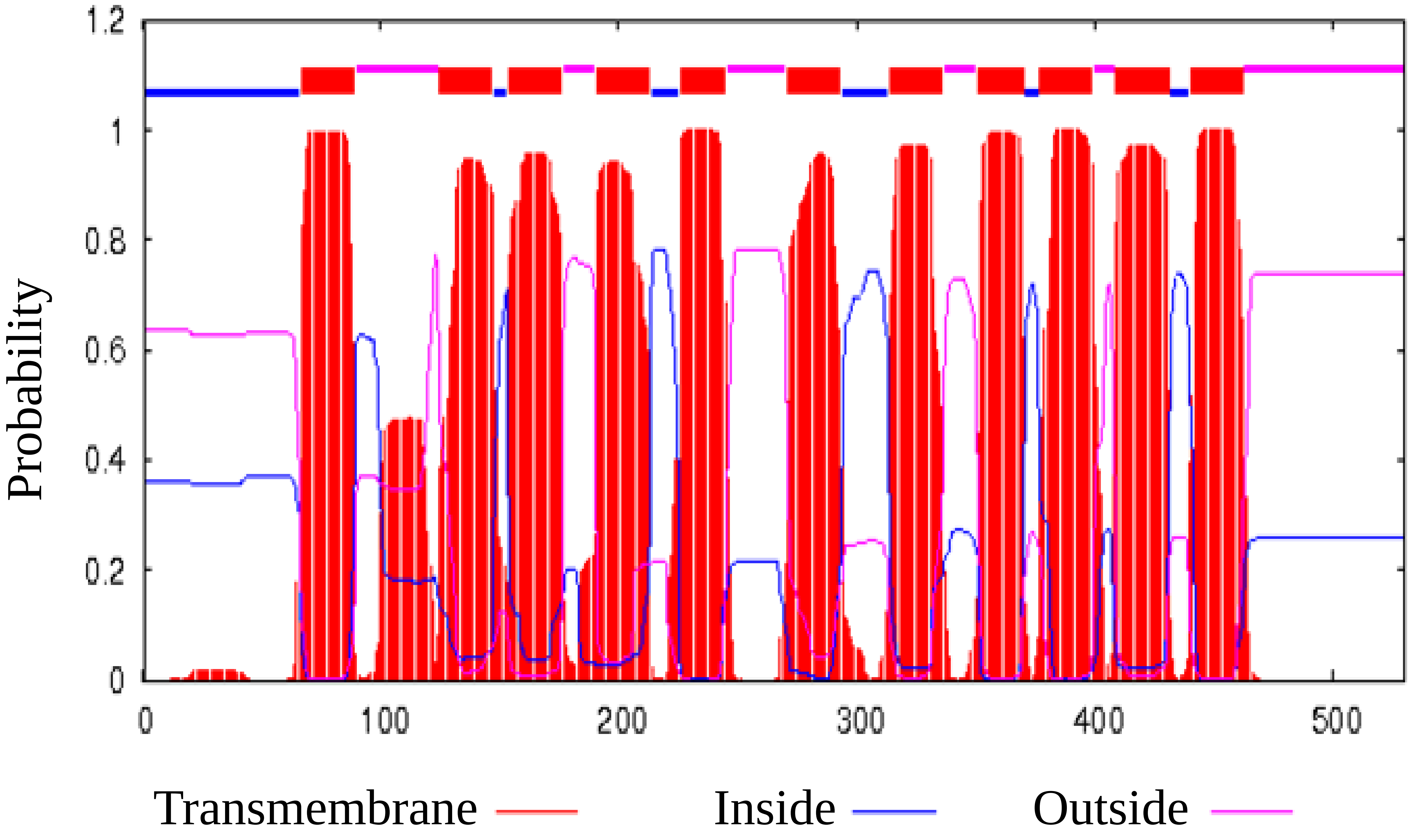

Supplementary Figure S2. Membrane-spanning model of CsNRT2.1. The position of N- and C-terminal domains of the protein are indicated by blue and pink lines. The predicted website was: TMHMM http://genome.cbs.dtu.dk/services/TMHMM-2.0.

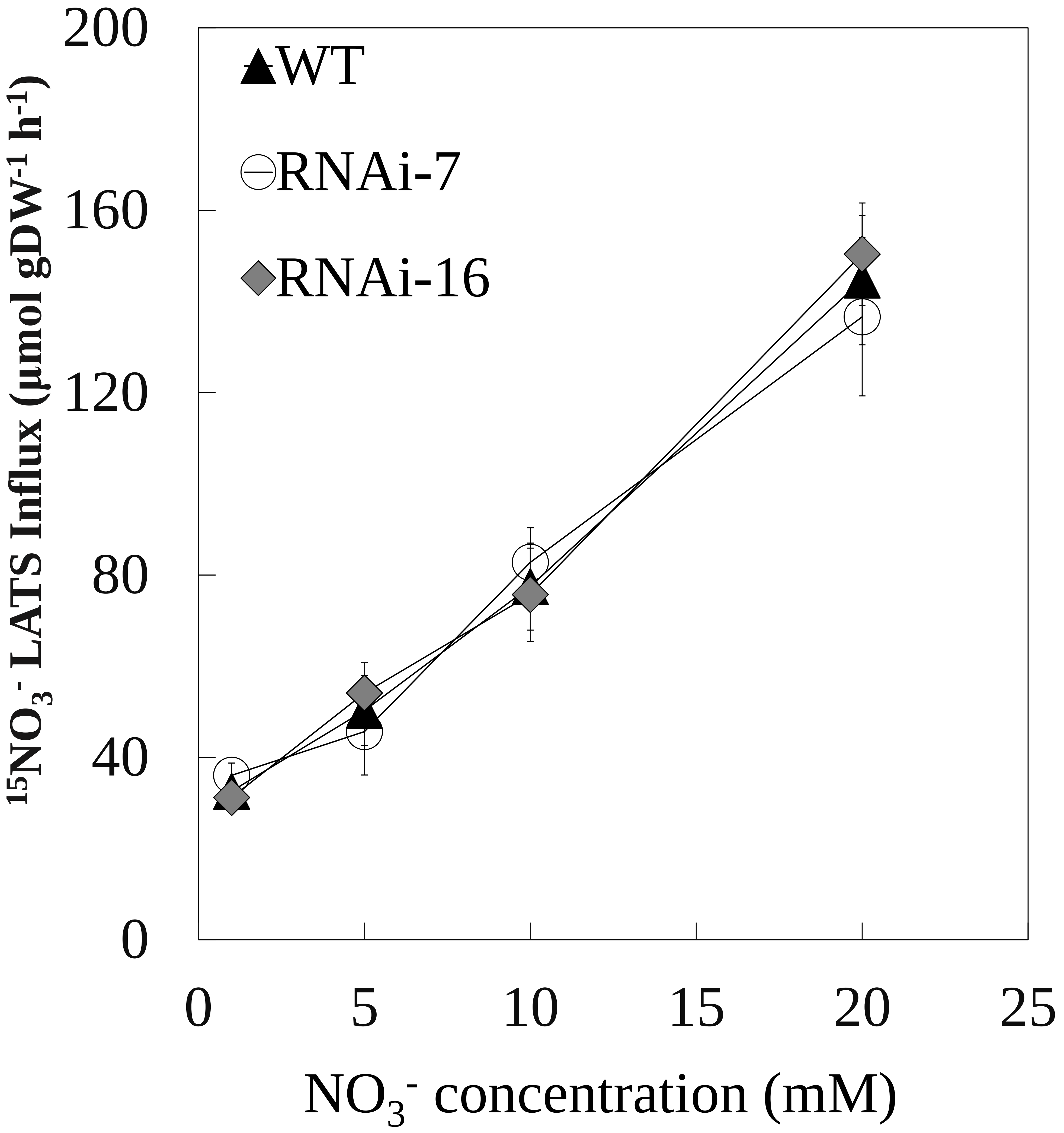

Supplementary Figure S3. Root $^{15}NO_3^-$ in flux in WT, RNAi-7 and RNAi-16. WT and RNAi plants were grown in 10 mM KNO_3 for 25 days and then deprived of N for 5 days. $^{15}NO_3^-$ influx was measured immediately from solutions containing various $^{15}NO_3^-$ concentrations. The values are means ± standard error (SE) of five biological replicates.

Supplementary Table S1. Primer sequences and their uses

Primers	Restriction sites and protect bases	Sequence (5'→ 3')	Used for
CsNRT2.1-F	————	ATGGGTGATGTTGAAGGTT	Cloning of *CsNRT2.1*
CsNRT2.1-R	————	TCAAACATGAGTAGGAGTTG TAT	
CsNRT2.1-RNAi-1-F	*BamH* I (GGATCC)	GGGATCCGAATCTAACCGCT ATGTAAC	*CsNRT2.1*-RNAi construct
CsNRT2.1-RNAi-1-R	*Spe* I (ACTAGT)	GACTAGTTGTTTCTGGGAGT ATCTTC	
CsNRT2.1-RNAi-2-F	*Asc* I (GGCGCGCC)	GGCGCGCCTGTTTCTGGGAG TATCTTC	
CsNRT2.1-RNAi-2-R	*Swa* I (ATTTAAAT)	ATTTAAATGAATCTAACCGCT ATGTAAC	
CsNRT2.1-GFP-F	*Spe* I (ACTAGT)	CGGACTAGTATGGGTGATGT TGAAGGTT	*CsNRT2.1*-GFP construct
CsNRT2.1-GFP-R	*BamH* I (GGATCC)	CGGGGTACCAACATGAGTAG GAGTTGTATTTG	
UBI-ep-F	————	CACCAAGCCCAAGAAGAT C	Internal controls in cucumber
UBI-ep-R	————	TAAACCTAATCACCACCAGC	
q-*CsNRT2.1*-F	————	CTTCTTCATTCTCATAATAC	qRT-PCR of *CsNRT2.1*
q-*CsNRT2.1*-R	————	TGCTCCGAGTCGACCGGTAA	
