# Supplementary material for: Knock-Down of CsNRT2.1, a Cucumber Nitrate Transporter, Reduces Nitrate Uptake, Root length, and Lateral Root Number at Low External Nitrate Concentration
Source: Front Plant Sci. 2018 Jun 1;9:722. doi: 10.3389/fpls.2018.00722 (PMC5992502; doi:10.3389/fpls.2018.00722)
Supplement: Supplementary file 2 [file Data_Sheet_2.DOCX]

**Supplementary data** Nucleotide and amino sequences of the CsNRT2.1.

CsNRT2.1 CDS:

ATGGGTGATGTTGAAGGTTCTCCAGGAAGCTCAATGCATGGAGTGACTGGAAGAGAACAAACCTTTGCATTCTCTGTAGCTTCCCCGATCGTCCCAACTGACACCACGGCTAAATTTGCATTACCGGTCGACTCGGAGCATAAAGCTAAGGTTTTCAGGATATGGTCTCTAGCCAACCCCCACATGAGAACCTTCCACCTTTCTTGGATTTCCTTCTTCACATGCTTTGTGTCCACCTTCGCAGCCGCCCCTCTCGTCCCCATCATTCGCGACAACCTCAACCTGACGAAAGTCGACATCGGGAACGCTGGAGTTACATCTGTTTCTGGGAGTATCTTCTCTCGTCTTGTGATGGGGGCTGTGTGTGACCTTCTTGGACCACGCTATGGATGTGCGTTTTTGATAATGTTGTCGGCTCCGACGGTGTTTTGTATGTCGTTTGTGTCGAATGCTGCTGGTTACATAGCGGTTAGATTCATGATAGGGTTTTCGCTAGCGACGTTTGTGTCGTGTCAGTATTGGATGAGTACAATGTTTAATAGTCAGATAATAGGGCTTGTGAACGGGACAGCAGCTGGGTGGGGTAATATGGGAGGAGGAGCAACTCAATTAATAATGCCATTGTTGTATGAGGTTATTCAGAGAGCTGGTGCTACTCCTTTTACTGCTTGGAGGATCGCTTTCTTCATTCCTGGTTTCCTTCATGTTCTTATGGGCATTTTGGTTTTGACTCTTGGTCAAGACTTGCCTGATGGTAACCTTGCTAGTCTTCAGAAGAAGGGAAACGTTGCCAAAGACAAATTCTCCAATGTCTTGTGGTATGCTGTGACCAATTATCGAACTTGGATCTTCGTCCTCCTCTATGGCTACTCCATGGGGGTCGAGCTTTCCACCGATAACGTCATTGCTGAATATTTCTACGATAGGTTCGATCTAAAGCTTCACACAGCGGGAATCATTGCAGCAACATTCGGGATGGCGAATCTAGTGGCTAGACCGTTTGGAGGGTATGCGTCTGACGTAGCAGCGCGATACTTTGGGATGAGAGGAAGGTTGTGGACACTATGGATCCTACAAACATTTGGGGGAGTGTTTTGCATGTGGCTGGGTAAAGCAACCAAGTTGCCAATAGCAATAGCGGCAATGATATTGTTCTCAATAGGAGCACAAGCAGCATGTGGAGCAACATTTGGTATAATTCCATTCATATCAAGGAGATCTCTGGGAATAATATCAGGACTAACAGGAGCTGGTGGAAACTTTGGAAGTGGTTTAACACAATTAGTATTCTTCTCAACAACAAAATACTCAACTGACACAGGATTGTTTTTGATGGGAATAATGATTGTTTGTTGCACTCTTCCAGTAACTTTGGTTCATTTCCCTCAATGGGGAAGCATGTTTCTACCACCAACTAAACATGTTGAAAAATCAACTGAAGAGTTTTATTATGGTTCTGAATGGACTGAGGATGAAAAGAAGAAAGGTTTGCATCAACAAAGTATTAAGTTTGCTGAGAACAGCAGATCTGAACGTGGTAGACGTGTTGCTTCAGCTCCAACTCCACCAAATACAACTCCTACTCATGTTTGA

CsNRT2.1 Protein:

MGDVEGSPGSSMHGVTGREQTFAFSVASPIVPTDTTAKFALPVDSEHKAKVFRIWSLANPHMRTFHLSWISFFTCFVSTFAAAPLVPIIRDNLNLTKVDIGNAGVTSVSGSIFSRLVMGAVCDLLGPRYGCAFLIMLSAPTVFCMSFVSNAAGYIAVRFMIGFSLATFVSCQYWMSTMFNSQIIGLVNGTAAGWGNMGGGATQLIMPLLYEVIQRAGATPFTAWRIAFFIPGFLHVLMGILVLTLGQDLPDGNLASLQKKGNVAKDKFSNVLWYAVTNYRTWIFVLLYGYSMGVELSTDNVIAEYFYDRFDLKLHTAGIIAATFGMANLVARPFGGYASDVAARYFGMRGRLWTLWILQTFGGVFCMWLGKATKLPIAIAAMILFSIGAQAACGATFGIIPFISRRSLGIISGLTGAGGNFGSGLTQLVFFSTTKYSTDTGLFLMGIMIVCCTLPVTLVHFPQWGSMFLPPTKHVEKSTEEFYYGSEWTEDEKKKGLHQQSIKFAENSRSERGRRVASAPTPPNTTPTHV
